# Identification of Top-Down Forces Regulating Cotton Aphid Population Growth in Transgenic Bt Cotton in Central China

**DOI:** 10.1371/journal.pone.0102980

**Published:** 2014-08-29

**Authors:** Peng Han, Chang-ying Niu, Nicolas Desneux

**Affiliations:** 1 Hubei Key Laboratory of Insect Resources Application and Sustainable Pest Control, Plant Science & Technology College, Huazhong Agricultural University, Wuhan, China; 2 French National Institute for Agricultural Research (INRA), Sophia-Antipolis, France; Ghent University, Belgium

## Abstract

The cotton aphid *Aphis gossypii* Glover is the main aphid pest in cotton fields in the Yangtze River Valley Cotton-planting Zone (YRZ) in central China. Various natural enemies may attack the cotton aphid in Bt cotton fields but no studies have identified potential specific top-down forces that could help manage this pest in the YRZ in China. In order to identify possibilities for managing the cotton aphid, we monitored cotton aphid population dynamics and identified the effect of natural enemies on cotton aphid population growth using various exclusion cages in transgenic Cry1Ac (Bt)+CpTI (Cowpea trypsin inhibitor) cotton field in 2011. The aphid population growth in the open field (control) was significantly lower than those protected or restricted from exposure to natural enemies in the various exclusion cage types tested. The ladybird predator *Propylaea japonica* Thunberg represented 65% of Coccinellidae predators, and other predators consisted mainly of syrphids (2.1%) and spiders (1.5%). The aphid parasitoids Aphidiines represented 76.7% of the total count of the natural enemy guild (mainly *Lysiphlebia japonica* Ashmead and *Binodoxys indicus* Subba Rao & Sharma). Our results showed that *P. japonica* can effectively delay the establishment and subsequent population growth of aphids during the cotton growing season. Aphidiines could also reduce aphid density although their impact may be shadowed by the presence of coccinellids in the open field (likely both owing to resource competition and intraguild predation). The implications of these results are discussed in a framework of the compatibility of transgenic crops and top-down forces exerted by natural enemy guild.

## Introduction

The widespread adoption of insect-resistant genetically modified (GM) Bt cotton has led to decreased use of chemical insecticides and enhanced biocontrol services provided by natural enemies in Northern China [1], [2]. The Yangtze River Valley Cotton-planting Zone (YRZ), which located in central China, is one of the largest cotton-growing regions nationwide [3]. In this region, several insect-resistant GM cotton cultivars, notably the transgenic cotton that combines the two genes *Cry1Ac* (Bt endotoxin) and *CpTI* (Cowpea Trypsin Inhibitor), have been widely adopted during the past decade [4]–[6]. The cotton aphid *Aphis gossypii* Glover (Hemiptera: Aphidiae), a pest not targeted by Bt endotoxin (as is the case with other aphids, e.g. see [7], [8]), is considered a secondary insect pest in the YRZ. Although cotton aphid populations have shown continuous decline in seasonal density in cotton fields in the past 15 years in Northern China [2], cotton aphid outbreaks may occur and reach economically damaging levels [1] owing to particular weather conditions (e.g. less rainfall during the aphid population-growth season) or pesticide resistance [9].

In agro-ecosystems, natural enemies play an important role in controlling arthropod pest populations [2], [10]. For example, Hawkins and Marino [11] reported that insect parasitoids caused the highest mortality among the biotic factors for many pest species (mortality compiled for 78 pest species). Symondson *et al.* [10] stressed the importance of generalist predators in regulating pest populations. Various studies have documented top-down forces regulating herbivore populations and crop biomass yield [12]–[15] and also identified key natural enemies of predators involved in pest suppression in specific crops [14], [16]–[18]. Indeed, it is crucial to characterize the guild of potential natural enemies capable of attacking targeted pest(s) for developing a sustainable Integrated Pest Management (IPM) program in any cropping system [19]–[21]. For example, identifying key natural enemies in a given ago-ecosystem may orient further research on how these natural enemies may be promoted to enhance biological control [22]–[25]. Therefore, studies documenting top-down forces in agro-ecosystems are crucial for developing effective IPM programs.

In Bt-cotton cropping systems in the YRZ, no systematic study has been carried out to characterize *A. gossypii* population dynamics and to identify the specific top-down forces that may help managing this pest in Bt cotton fields. In the present study, using various types of natural enemy exclusion cages and artificially released aphid populations, we aimed to (i) monitor aphid population dynamics in open field, (ii) assess specific effects of natural enemies on *A. gossypii* population dynamics, and (iii) identify the key natural enemies of *A. gossypii* in Bt cotton. The results of the present study will help optimize integrated management of *A. gossypii* in Bt-cotton cropping systems in central China.

## Materials and Methods

### Cotton field and aphid colony

Experiments were conducted during the summer of 2011 at Ezhou experimental station (Huazhong Agricultural University), Ezhou, Hubei province, China (114.7 E, 30.3 N). The GM cotton cultivar CCRI41 (Zhongmian 41) which produces insecticidal proteins Cry1Ac (*Bt* endotoxin) and CpTI (Cowpea trypsin inhibitor) [4], [26] was used during the study. The CCRI41 seeds were provided by the Institute of Cotton Research of Chinese Academy of Agricultural Sciences (CAAS), Anyang, China. Seeds were sowed on April 27^th^ in a 1.5-ha cotton field with 1-m spacing between rows and the cotton was cultivated using standard agronomic techniques except that no pesticides were applied. The field had been used for cotton cultivation for several years. The area surrounding the field consisted of mainly cotton (55%), rice (30%), sweet potato (10%), other minor cropping plants, and natural habitats.

Naturally occurring *A. gossypii* were collected in May from a cotton field at Huazhong Agricultural University (Wuhan, China) which had been cropped without insecticide applications. These aphids were used to establish a colony in the laboratory (on cotton) at the university and used as the source of aphids for infesting the plants during the field study.

### Experimental setup

Four different degrees of predator exclusion were tested using various exclusion designs in the Bt cotton field: (i) Exclusion cages with 530×530 µm openings in which aphids were fully protected from all insect natural enemies. (ii) Restriction cages with 3×3 mm openings in which aphids were partially protected. This size of openings restricted entry by large predators i.e. Coccinellids, but allowed small predators to enter [13], [27]. (iii) Sham cages built with 530×530 µm mesh netting but included a 40 cm high opening in the middle and the bottom respectively (modified from [15]). This treatment was used to assess possible disruptive effect of caging (e.g. mesh, wood sticks, etc.) on the activity of natural enemies and aphid population growth within the plots. (iv) No cage, a completely open area (named “open field” hereafter), which used four wood sticks standing upright into the ground and a tape surrounding them as guidance for sampling range and plot size and position.

**Figure 1:**
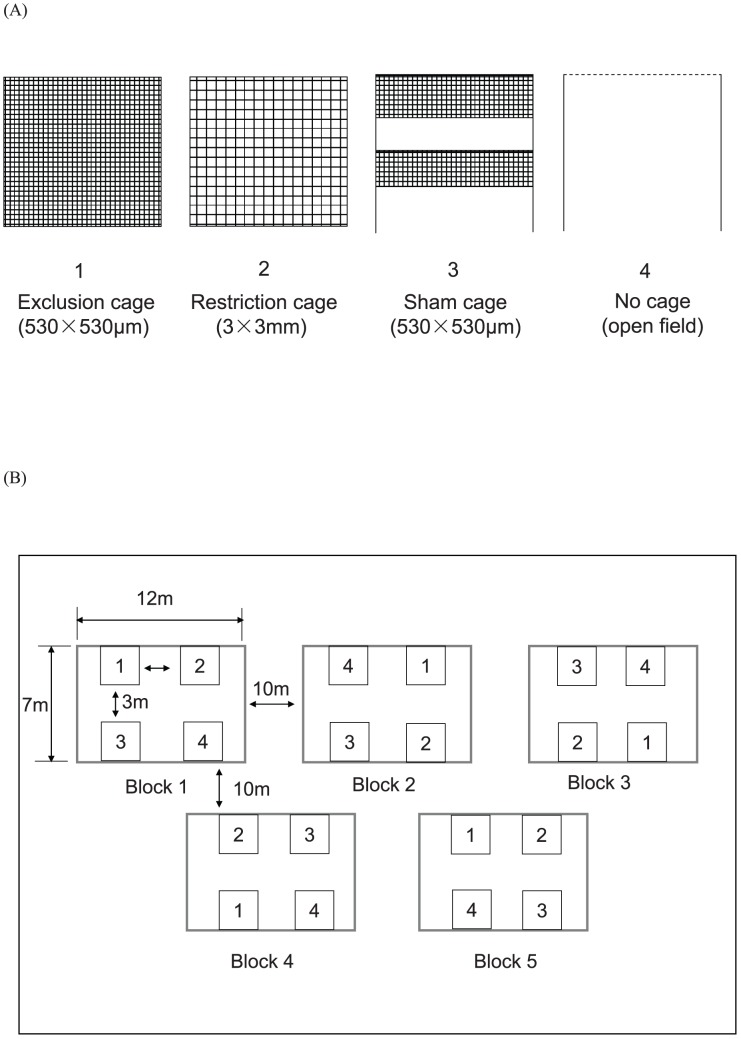
Design of the field study. (**A**) The four different cage treatments, i.e. natural enemy exclusion degree, in the field study; Exclusion cages: prevented natural enemy (predators and aphid parasitoids) movement, Restriction cage: prevented predator movement but allowed aphid parasitoids to colonize the plants, Sham cage and No cage: allowed free access to the plants for all natural enemies. (**B**) Within- and among block design: the distance among treatments within a block was 3 m, and among blocks was 10 m. The experimental cotton field was 70 m×30 m.

The four different treatments were established on July 28^th^ (Fig. 1a) using a completely randomized block design (Fig. 1b). The distance between treatments inside each block was 3 m and between blocks was 10 m. The field cages were made of wood frames (2×2×2 m, length×width×height) covered by fine nylon mesh netting with openings of 530 µm or 3 mm according to the various designs used, see above). Four plants were enclosed in each cage with a distance of 1 m between plants. We used 2×2×2 m cages because the cotton cultivar used could grow up to 1.8 m height and 1 m width during the season [4], [5]. One side of each cage was equipped with a zipper to enable sampling.

Prior to the artificial aphid infestation, any resident aphids and other insects were removed by hands, brushes and mouth aspirators in all of the cages and plants in the open field plots (20 plots total). On July 28^th^, ten aphids were released on each plant of the four different treatments. Aphids were placed on the highest central leaf of the plants using a camel's hair brush. From August 4^th^ to Sept 30^th^, all arthropod pests and natural enemies on the four plants within each plot were recorded and identified to family or species level. In the case of aphid parasitoids, the non-emerged parasitoid mummies (pupae stage of the parasitoid) were counted (with black- and tan-colored mummies assigned to the Aphelinidae and Aphidiinae parasitoid families, respectively). The field survey was carried out on a weekly basis (every 7–8 days) from noon to 6 pm for each date of survey. Mummy samples were collected from the various plots during the course of the study (mainly from Aug 20^th^ to Sept 7^th^ when parasitoid densities were at high levels) for further identification of parasitoids using appropriate identification keys by [28]–[31]. The collected mummies (n = 119) were brought back to the laboratory and placed in Petri dishes in a Climatic Chamber (25°C, 65% RH and 16∶8 h/L∶D) until parasitoid adults emerged.

### Statistical analysis

We tested the effect of predator exclusion degree (factor: cage type), as well as the effect of the date (factor: date) on the aphid counts and on numbers of main natural enemies recorded (see below) using a generalized linear model based on a Poisson distribution and a log-link function (Proc Genmod in the SAS statistical package, SAS Institute, NC, USA).

## Results

Overall, three dominant arthropod guilds were identified during the surveys: (i) pest insects, (ii) natural enemies of *A. gossypii* and (iii) omnivorous insects (Table 1). *Aphis gossypii* accounted for 85.1% of total pest insects recorded; the other three main pest species were the leafhopper *Empoasca biguttula* Shiraki, the whitefly *Bemisia tabaci* Gennadius and the common cutworm *Spodoptera litura* Fabricius (Lepidoptera: Noctuidae). The natural enemy guild was largely dominated by the aphid parasitoids which accounted for 76.7% of all natural enemies recorded during the study. Aphidiines (tan-colored mummies) were most commonly observed; only 3 Aphelinidae mummies were found during the study. The parasitoids identified (i.e. those emerged from mummies brought back to the laboratory) were primarily *Lysiphlebia japonica* Ashmead and *Binodoxys (Trioxys) indicus* Subba Rao & Sharma (51.8% and 37.7% of samples collected, respectively). Two other species were also identified at lower rates: *Aphidius gifuensis* Ashmead and *B. near communis* (8.8% and 1.8%, respectively). Coccinellids represented 11.2% of all natural enemies observed, with *Propylaea japonica* Thunberg being the dominant species belonging to this group of predators (65.03%). *Harmonia axyridis* Pallas (20.04%) and *Coccinella septempunctata* Linnaeus (14.92%) were also observed as less common coccinellid species. The other natural enemies belonged to the syrphid, spider and lacewing predator groups. Omnivorous insects were also observed, mainly Hemipteran piercing-sucking bugs belonging to the Miridae, Nabidae and Anthocoridae families.

**Table 1:** Dominant arthropods, per guild, found during the surveys.

Guild	Taxonomy	Total counts	Percentage within guild (%)
Pest insects	*Aphis gossypii* Glover	30611	85.1
	*Empoasca biguttula* Shiraki	1693	4.7
	*Bemisia tabaci* Gennadius	2727	7.6
	*Spodoptera litura* Fabricius	924	2.6
Natural enemies	Coccinellids^a^	449	11.2
	Aphid parasitoids (Aphidiines)	3081	76.7
	Syrphidae	83	2.1
	Araneae^b^	60	1.5
	Chrysopa (lacewings)	52	1.3
Omnivorous insects	Hemiptera (bugs)^c^	124	

Total counts of dominant arthropods per guild in the experimental blocks during the field survey from August 4^th^ to September 30^th^, 2011, in Ezhou (China).

a mainly *Propylaea japonica* Thunberg (292, 65.03%), *Harmonia axyridis* Pallas (90, 20.04%) and *Coccinella septempunctata* Linnaeus (67, 14.92%).

b mainly *Erigonidium graminicolum* Sundevall.

c mainly from Miridae, Nabidae and Anthocoridae families.

**Figure 2:**
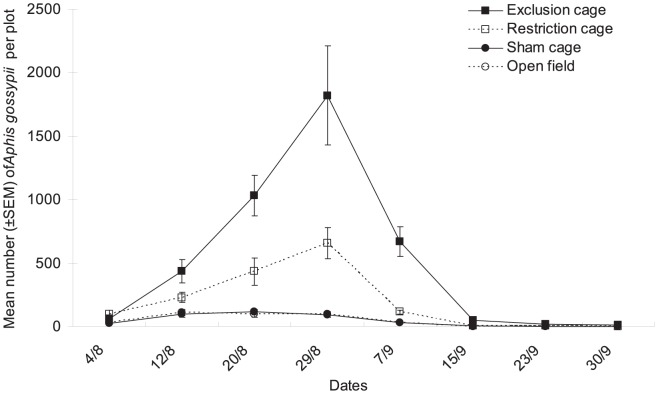
Cotton aphid population dynamics. Mean numbers (±SEM) of *A. gossypii* per plot in the various natural enemy exclusion treatments from early August to end of September in Bt cotton in Ezhou (China).

*Aphis gossypii* densities we recorded differed significantly among cage types (Fig. 2, cage type factor: χ^2^ = 12.20, df = 3, *P* = 0.007) and as function of the dates when the aphid populations were surveyed during the season (date factor: χ^2^ = 17.73, df = 7, *P* = 0.013). The two factors did not interact significantly when analyzing aphid counts (χ^2^ = 20.07, df = 21, *P* = 0.521). More aphids were found in exclusion cages and restriction cages than in sham cages or open field plots. There was a 180-fold aphid population growth by August 29^th^ from the initial aphid count at the July 28^th^ release in the exclusion cages, whereas there was only a 10-fold aphid population increase in the sham cages and open field plots. Sham cages and open field plots showed no difference in aphid numbers during the course of this study (Fig. 2).

**Figure 3:**
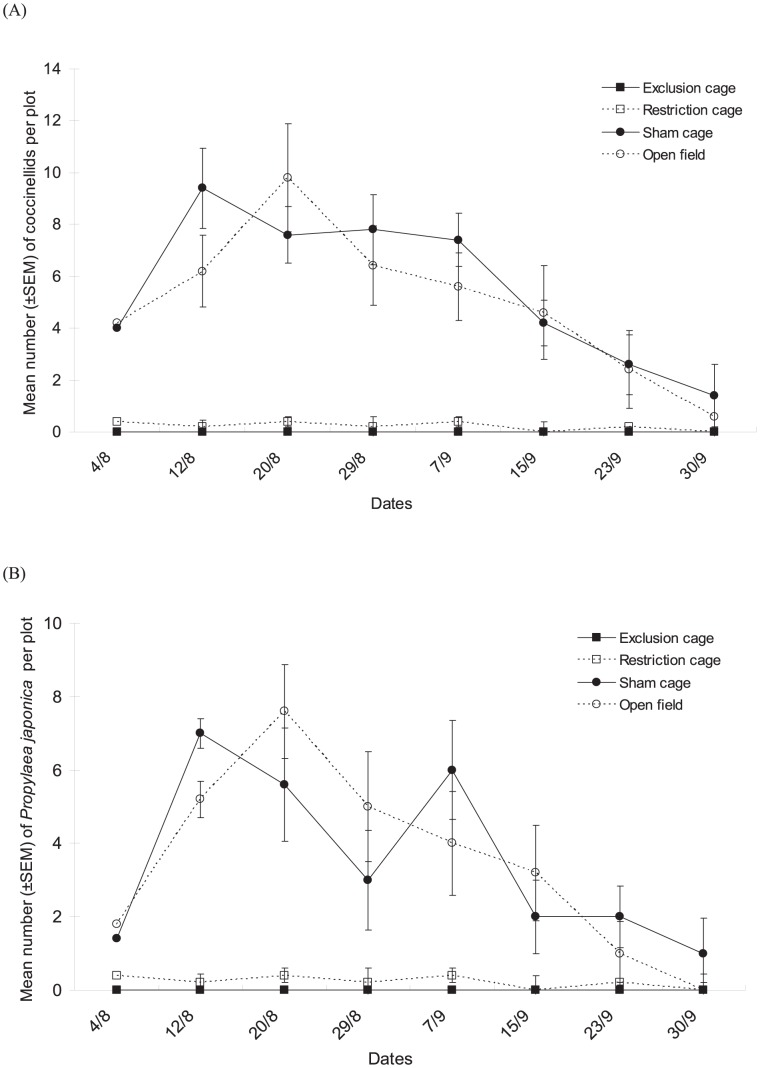
Population dynamics of coccinellid predators. Mean numbers (±SEM) of (**A**) all Coccinellids and (**B**) *P. japonica* per plot in the various natural enemy exclusion treatments from early August to end of September in Bt cotton in Ezhou (China).

The numbers of coccinellids recorded also differed significantly among cage types (Fig. 3A, cage type factor: χ^2^ = 18.20, df = 3, *P*<0.001) and among dates of sampling (date factor: χ^2^ = 19.52, df = 7, *P* = 0.007). There was no significant interaction between the two factors (χ^2^ = 19.87, df = 21, *P* = 0.134). Many more coccinellids were recorded in sham cages and open field plots than in exclusion cages and restriction cages; however no difference in coccinellids was observed between sham cages and open field plots. *Propylaea japonica* was the dominant species among the Coccinellidae family during the survey (Fig. 3B). The counts for this species followed the same trends as were observed for the coccinellid group as a whole: more *P. japonica* were found in sham cages and open field plots (significant cage type factor: χ^2^ = 19.00, df = 3, *P*<0.001, and date factor: χ^2^ = 19.89, df = 7, *P* = 0.006, no significant interaction: χ^2^ = 23.12, df = 21, *P* = 0.333).

**Figure 4:**
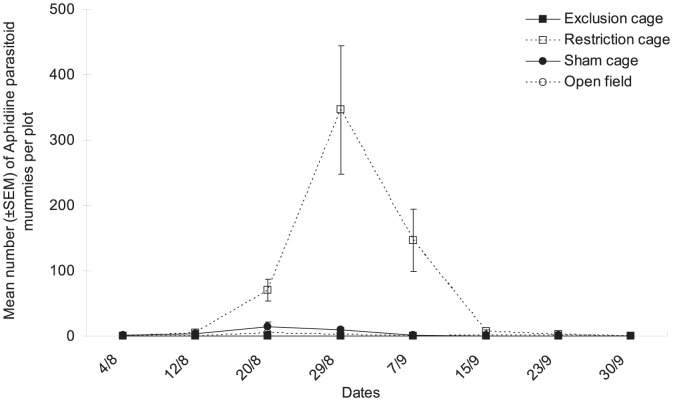
Population dynamics of aphid parasitoids. Mean numbers (±SEM) of Aphidiine per plot in the various natural enemy exclusion treatments from early August to end of September in Bt cotton in Ezhou (China).

The numbers of Aphidiine mummies differed significantly between cage types (Fig. 4, cage type factor: χ^2^ = 8.91, df = 3, *P* = 0.031) and dates (date factor: χ^2^ = 19.03, df = 7, *P* = 0.008), but the two factors did not interact significantly overall (χ^2^ = 22.89, df = 21, *P* = 0.274). Overall, many more Aphidiine parasitoids were found in restriction cages than in the other three cage treatments on Aug 20^th^, Aug 29^th^ and Sept 7^th^ (Fig. 4); the parasitoid density increased markedly by 30- to 40-fold beginning Aug 12^th^ and reached a peak on Aug 29^th^.

**Figure 5:**
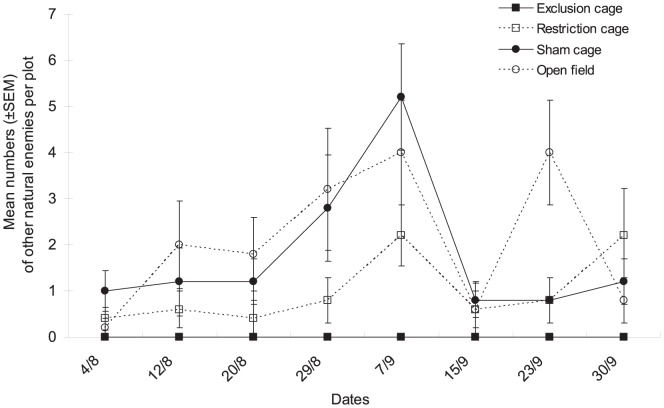
Population dynamics of other natural enemies. Mean numbers (±SEM) of other natural enemies per plot in the various natural enemy exclusion treatments from early August to end of September in Bt cotton in Ezhou (China).

The numbers of other natural enemies differed significantly among cage types as well (Fig. 5, cage type factor: χ^2^ = 10.16, df = 3, *P* = 0.017) and dates (date factor: χ^2^ = 18.61, df = 7, *P* = 0.046), no significant interaction was observed between the two factors (χ^2^ = 25.67, df = 21, *P* = 0.458).

## Discussion

Our study demonstrated the contribution of natural enemies (predators and parasitoids) on cotton aphid population growth in Bt+CpTI cotton field. In the absence of predators and parasitoids resulting from exclusion cages, cotton aphid populations increased up to maximum of 180-fold from aphid density at the initial release date, while in the presence of natural enemies (open field plots or sham cages) aphid populations showed a maximum 10-fold increase. These major differences in aphid population dynamics show the importance of top-down forces on this pest infesting Bt cotton. We identified the coccinellid *P. japonica* and the Aphidiine parasitoids as the predominant natural enemies in the cotton field, with distinct but additive effects on cotton aphid population growth. The best control of aphid populations was obtained when both natural enemy types had access to the aphids in open field plots or sham cages.

The coccinellid *P. japonica* proved to be an important natural enemy for suppressing cotton aphid population growth in Bt cotton fields in the YRZ in China. *Propylaea japonica* is a well-known predator of *A. gossypii* [32], [33] and its life history characteristics and phenology make it a good candidate biocontrol agent for management of the aphid in Bt cotton. This predator colonizes cotton fields early in the cotton seedling stage, at the same time as the aphid population starts infesting the cotton field. Being a generalist predator, it can feed on a variety of prey including spider mites, thrips, whites flies and other small species [34], [35], including those observed during our study (e.g. whiteflies and leafhoppers, see Table 1). Alternative prey can help promote establishment of predators early in the season when the targeted pest is scarce (e.g. see [36]). Therefore, *P. japonica* can effectively delay the establishment and subsequent population growth of aphids early in the growing season. Such characteristics often make, generalist predators useful in the strategy of conservation biological control (e.g. see [10], [37], [38]).

The Aphidiine parasitoids, mainly *L. japonica* and *B. indicus*, were also found to suppress cotton aphid population growth. In the restriction cages, when coccinellid predators did not have access to the aphid populations, the parasitoids reduced aphid peak population by nearly 2/3 (see aphid densities in exclusion cages vs. restriction cages, Fig. 2). However, Aphidiines alone could not totally prevent aphid population growth, as aphid density reached ∼600 aphids per plot by Aug 29^th^. In these restriction cages there was a rapid early season aphid population growth because predators known to limit pest population increase early in the season were excluded [10]. However, as aphid density increased in these plots, aphid parasitoid adults were attracted and this resulted in abundant parasitized mummies in the following weeks. When predators were present (in the sham cages and open field plots) the parasitoid populations remained at low densities throughout the season, either because of possible intraguild predation [39], [40] of parasitoid mummies by coccinellids (e.g. see [41]), or through resource competition of aphid parasitoids (aphids) with the generalist predators in the plots [42], [43]. In this instance, the aphid parasitoids may help reduce aphid densities primarily when aphid populations have already reached a certain density. Previous surveys of natural enemies of cotton aphid carried out in different regions of China produced variable collections of species records. Sun *et al.* [34] reported that the predator guild in cotton fields near Beijing (Xibeiwang) was dominated by *Chrysoperla sinica* Tjeder, *P. japonica*, various spiders and *Orius minutus* L. The same authors also reported that *Lysiphlebia japonica* was the dominant aphid parasitoid; the parasitoid guild in Xibeiwang region may be similar to the one recorded in YRZ. Zhou *et al.* [44] reported similar findings to ours as *L. japonica* and *P. japonica* were dominant in Hebei province. However, in contrast to our results, they reported very low biodiversity in coccinellid species (we found that *H. axyridis* and *C. septempunctata* were well represented in our plots) and that *C. sinica* and heteropteran predators (mainly *O. minutus* and the mirid predator *Campylomma diversicornis* Reuter) were quite abundant (as much as coccinellids). In the Xinjiang region, Xu *et al.* [45] conducted surveys on predators and found them, in order of importance in terms of density, coccinellids>spiders>lacewings>heteropteran predators. These contrasting results among geographic regions highlight the need to identify the specific natural enemies at play in a given region when developing conservation biological control programs.

When examining the aphid parasitoid group, it is worth mentioning that Aphelinid parasitoids were nearly absent from the field (as reported in other agro-ecosystems, e.g. in *Brassicae* crops [46], [47]). When considering Aphidiine parasitoids, *L. japonica* proved to be a key natural enemy of cotton aphid in Northern China [44], [48]. This species is also a natural enemy of phylogenetically closely related aphid species [49] e.g. the soybean aphid *Aphis glycines* Matsumura in Japan and Indonesia [50] and the brown citrus aphid *Toxoptera citricida* Kirkaldy [51], [52]. Several species from the *Binodoxys* genus are known to efficiently attack *A. gossypii* [31], [53], [54] and *B. indicus* may be an important natural enemy of this aphid pest in the YRZ region as well as other regions not extensively surveyed.

Ecological compatibility of GM crops and natural enemies is a key issue for implementing biological control programs within GM cropping systems [55]–[57]. Previous studies suggested that Bt+CpTI cotton might not affect population dynamics of natural enemies [34], [45]. No effect was observed on the fitness of *P. japonica* when fed with *A. gossypii* on Bt cotton ([33], [58], but see [59]). In addition, aphid parasitoids may not be exposed to Bt toxins [7], [8]. However, they can be negatively affected by Trypsin Inhibitors [60] e.g. CpTI. Zhou *et al.* [44] reported a 44% decrease in *L. japonica* population density in Bt-CpTI cotton fields. Although we did not carry out a formal comparison between non Bt and Bt cotton cultivar, we highlighted a strong top-down effect on cotton aphid populations. Therefore natural enemies, as a whole group, are effective in limiting aphid population growth in Bt-CpTI cotton fields.

Our study demonstrated the importance of the top-down force exerted by natural enemies, mainly coccinellids and Aphiddiine parasitoids, on cotton aphid in Bt cotton field in China. However, the relative strength of top-down vs. bottom-up forces on *A. gossypii* still needs to be studied in order to develop IPM including such forces in a sustainable and comprehensive way, especially since various studies have already identified the importance of bottom-up forces (e.g. fertilization regime) on herbivore population dynamics [61]–[64]. Developing such optimized IPM would help manage secondary pests that may show population outbreaks in Bt cotton since its wide spread adoption in China. For example, *S. litura* larvae were found in relatively high density during our surveys and this species can cause considerable damage to cotton crops. This finding is consistent with the reported low susceptibility of this pest species to current Bt cotton cultivars [3]. Secondary pests may promote applications of insecticides in Bt cotton with potential associated multiple negative effects on human health and non-target organisms [65]–[67]. Highly selective chemical pesticides may be required at times [68], [69] but limiting the application of pesticides and promoting more sustainable pest management strategies should be prioritized. For example, optimized IPM may aim at combining biocontrol agents as top-down force [70], [71] with bottom-up forces like fertilization regimes and/or cultural practices [13], [72]–[75] for efficient management of pests. In addition, the sustainable use of GM crops can lead to drastic reduction in pesticide usage at the wide scale [2]; developing optimized IPM in Bt crops such Bt cotton would help capitalize on the benefits provided by transgenic methods in cropping systems.
